# Citalopram *in vitro* metabolism in a Beagle dog: A role for CYP2D15 in the production of toxic didesmethylcitalopram?

**DOI:** 10.17221/65/2022-VETMED

**Published:** 2023-04-28

**Authors:** Bertrand Rochat, Erik Paus, Cedric Maitre, Pierre Baumann

**Affiliations:** ^1^Lausanne University Hospital and University of Lausanne, Lausanne, Switzerland; ^2^Department of Education and Research, University of Lausanne, Lausanne, Switzerland; ^3^School of Pharmaceutical Sciences, University of Geneva, Geneva, Switzerland, and Institute of Pharmaceutical Sciences of Western Switzerland, University of Geneva, Geneva, Switzerland; ^4^PharmaciePlus Franches-Montagnes, Saignelégier, Switzerland; Bertrand Rochat, Erik Paus and Pierre Baumann contributed equally to this work

**Keywords:** cytochrome P-450, liver microsomes, stereoselectivity

## Abstract

After administration of the serotonergic antidepressant citalopram (CIT) to Beagle dogs, the dogs may experience severe convulsive attacks in relation to the considerably higher plasma concentrations of the metabolite didesmethyl-CIT (DDCIT), when compared to those in humans medicated with CIT. This pilot study aimed at determining the role of cytochrome P-450 (CYP450) isozymes in the *in vitro* metabolism of CIT to desmethyl-CIT (DCIT), and of DCIT to DDCIT in the liver microsomes of a single Beagle dog. Incubations with racemic CIT or DCIT reveal a high-affinity enzyme with K_m_ between 0.3 μM and 1.4 μM for *S*- and *R*-DCIT and *S*- and *R*-DDCIT productions, respectively. In comparison to human enzymes, the intrinsic clearance values of this high-affinity enzyme are between 15 μl/(min × mg of protein) and 52 μl/(min × mg of protein), i.e., very high. *In vitro* experiments with inhibitors suggest that CYP2D15, which shows an analogy with human CYP2D6, is by far the main CYP450 isozyme involved in the production of DCIT and DDCIT, whereas CYP3A12 and CYP2C21/41 showed a weak implication. These observations partly explain why, in humans, the plasma concentrations of the toxic DDCIT are considerably lower than those observed in dogs, after administration of CIT.

The widely introduced antidepressant citalopram (CIT), a selective serotonin reuptake inhibitor (SSRI), is a racemic mixture of *R*-citalopram (*R*-CIT) and *S*-citalopram (escitalopram; *S*-CIT). The inhibition of serotonin [5-hydroxytryptamine (5-HT)] reuptake in the synaptic cleft by CIT shows stereoselectivity and it is primarily due to *S*-CIT, which is also available as an antidepressant (Hyttel et al. 1992; Hyttel 1993). In humans, CIT is mainly metabolised to the demethylated metabolites *N*-desmethyl-CIT (DCIT) and *N*-didesmethyl-CIT (DDCIT) by the cytochrome P-450 (CYP450) isoforms CYP2C19 and CYP3A4 and, to a minor degree, by CYP2D6 (Kobayashi et al. 1997; Rochat et al. 1997; Olesen and Linnet 1999). In addition, MAO-A and MAO-B contribute stereoselectively to the formation of the propionic acid metabolite (CIT-PROP) (Rochat et al. 1998), but this metabolite is also formed by CYP2C19 (Rudberg et al. 2009). Both DCIT and DDCIT (Hyttel et al. 1992; Hyttel 1993) are also SSRIs, but their potency as serotonin reuptake inhibitors is lower than that of *S*-CIT, and their concentrations in blood, especially those of DDCIT, are considerably lower than those of the parent compounds in CIT treated patients. In steady-state conditions, DDCIT concentrations are often below the level of quantitation (< 3 ng/ml or 10 nM) suggesting that DDCIT production is a minor pathway in humans (Rochat et al. 1995; Sidhu et al. 1997; Rasmussen et al. 1999; Carlsson et al. 2001), even in patients treated with 60–360 mg/day CIT (Le Bloc’h et al. 2003). Both CIT and *S*-CIT are relatively well tolerated, but dose restrictions were introduced for both drugs after the observation that they increase the QTc-interval and the risk for torsades de pointes (TdP) in patients. Therefore, they are classified in risk group 1 (www.crediblemeds.org). According to another classification, they belong to the category Class B, which means that there is an associated risk of arrhythmia and a “possible TdP risk” (Fanoe et al. 2014).

Already in 1982, studies carried out by the manufacturer showed that the serotonergic antidepressant CIT can elicit severe convulsive attacks in dogs, but, in that study, no electrocardiographic changes were observed (Boeck and Overo 1982).

There are important species differences in the metabolism of CIT, as pharmacokinetic studies have demonstrated a considerably higher formation of DDCIT in dogs than in humans after administration of CIT (Fredricson Overo 1982; Matsui et al. 1995), as measured in the blood using non-stereoselective analytical procedures. The mean elimination half-life of CIT, DCIT and DDCIT in dogs reached 9.6 h, 8.1 h and 27.8 h, respectively (Matsui et al. 1995). In Beagle dogs, DDCIT concentrations > 1 000 nm (324 ng/ml) were considered responsible for the lengthening of the QT interval and the induction of arrhythmia. This adverse effect seems to be particularly pronounced in situations when both CIT and DDCIT present high levels in the blood of dogs (Fredricson Overo 1982; Fredricson Overo 1989; Fredricson Overo and Hojelse 1994).

However, there are, as yet, no studies on the role of CYP450 isozymes in the *in vitro* metabolism of CIT in dog liver microsomes. This pilot study was designed to explain the high formation rate of DDCIT in this species. It is to be considered that there are important inter-species differences between CYP450 isoforms. This results in characteristic denominations for each species. As an example, some authors proposed the following equivalences, based on some similarities and despite important differences: Humans: 1A2 ~ dog: 1A2; humans: CYP2C19 ~ dog: CYP2C21; humans: CYP2D6 ~ dog: CYP2D15; CYP3A4 ~ dog: CYP3A12 (Martignoni et al. 2006).

## MATERIAL AND METHODS

### Animal welfare and ethics statement

All procedures involving animal tissues were done in strict accordance with the Helsinki Declaration (2000) and with the Swiss Governmental directives concerning scientific research.

### Chemicals

The racemates of CIT hydrobromide, DCIT hydrochloride and DDCIT l-tartrate, and their *S*- and *R*-enantiomers were obtained from Lundbeck (Copenhagen, Denmark). These compounds were dissolved in a 0.1 M phosphate buffer (pH 7.4) at a concentration of 1 mg/ml base as stock solutions and in a 0.1 M phosphate buffer (pH 7.4) at concentrations ranging from 1 ng/μl to 100 ng/μl, and stored at –20 °C. The internal standard *S*-alprenolol was purchased from Sigma (Basle, Switzerland), dissolved as a stock solution in methanol at a concentration of 1 mg/ml, and in 0.1 M hydrochloric acid at 2 ng/μl and 10 ng/μl as working solutions, and stored at –20 °C until use.

The inhibitors of CYP3A12 (ketoconazole), CYP2D15 (quinidine), CYP2C21/41 (omeprazole), CYP2E1 (4-methylpyrazole), CYP2C9 (sulphaphenazole) and CYP1A2 (furafylline) were supplied by Sigma (Basle, Switzerland). The following solutions were prepared: 5 μM ketoconazole in a 0.1 M phosphate buffer (pH 7.4), 0.2 mM quinidine in a 0.1 M phosphate buffer (pH 7.4)/DMSO (90/10), 1 mM omeprazole in a 0.1 M phosphate buffer (pH 7.4), 2 mM 4-methylpyrazole in a 0.1 M phosphate buffer (pH 7.4), 0.5 mM sulphaphenazole in a 0.1 M phosphate buffer (pH 7.4)/DMSO (98/2), and furafylline in a 0.1 M phosphate buffer (pH 7.4)/DMSO (98/2). Triethylamine (Lot No. 90340) and methanol were purchased from Fluka (Buchs/SG, Switzerland), and citric acid (Lot No. A016250801) from Acros Organics (Geel, Belgium).

### Preparation of dog liver microsomal fractions and incubations

The liver was kindly provided by Lundbeck (Copenhagen, Denmark). It came from a four-year old Beagle dog, of undefined sex, which was drug-naive for about half a year.

The microsomal fractions were prepared as previously described (Dayer et al. 1987) and stored at –80 °C until use. The proteins were assayed by a colorimetric procedure (Lowry et al. 1951). The total protein content was not measured, as Dayer et al. (1987) reported: Protein content of human liver microsomes: 2.21–5.66 mg/ml; spectrally measured P450: 0.099–0.366 nmole P450/mg protein. The incubations of the dog liver microsomes and kinetic studies were carried out using procedures described earlier (Rochat et al. 1997; von Moltke et al. 2001). Prior to use, after the microsomal preparations were thawed at 4 °C for one hour, 100 μg of the dog liver microsomal protein in a 0.1 mM KH2PO4 buffer (pH 7.4) were pre-incubated for 15 min at 37 °C with 20 μl of a nicotinamide adenine dinucleotide phosphate (NADPH) generating system (0.33 mM NADP, 5 mM isocitrate, 5 mM MgCl_2_, 1 unit isocitrate dehydrogenase-type IV) solution. Different concentrations of the CIT or DCIT substrates were added to the pre-incubated mixture to a final volume of 250 μl and incubation was carried out for 1 h at 37 °C. Incubation was stopped by the addition of 750 μl of 0.250 M NaOH at 4 °C and was stored at –80 °C until extraction.

For the measurement of K_m_ and V_max_, incubations were performed with CIT and DCIT concentrations ranging from 1 to 1 000 μM (von Moltke et al. 2001). Per situation, five and three incubations were carried out with CIT and DCIT, respectively. Incubations were performed with 100 μg of the proteins and 20 μl of the NADPH generating system using 0.5, 1, 5, 10, 50, 100, 200, 500, 750, 1 000 μM of CIT or DCIT as the substrate. Two incubations in triplicate per substrate were performed.

For the inhibition study, the stereoselective metabolism of CIT and DCIT to DCIT and DDCIT, was investigated in the presence of 1 μM of ketoconazole (Rochat et al. 1997; Kuroha et al. 2002), 100 μM of quinidine (Chauret et al. 1997) or 10 μM of omeprazole.

### Extraction and liquid-chromatographic conditions

The extraction conditions were slightly modified compared to those previously reported (Rochat et al. 1997). Due to interference, benzoctamide and desmethylbenzoctamide previously proposed as internal standards (Kosel et al. 1998) were replaced by *S*-alprenolol. For the extraction of incubation mixtures, 500 μg of *S*-alprenolol, 50 μl of 1 M NaOH and 1.5 ml of heptane-isoamyl alcohol (98.5 : 1.5 v/v) were added to the mixture which was then shaken for 15 min and centrifuged at 3 300 *g* for 8 min at 8 °C. The organic layer was transferred to a wide opening crimp vial and was evaporated to dryness under a stream of nitrogen at 40 °C. The compounds were then dissolved in 150 μl of the mobile phase and finally transferred into an injection tube.

This procedure allowed the extraction of the enantiomers of CIT, DCIT and DDCIT, but not of CIT-*N*-oxide and CIT-PROP. Generally, 100 μl of the contents of the injection tube were injected, except when the CIT or DCIT concentrations exceeded 100 μM. In this case, only 10 μl of the contents of the injection tube were injected to allow the quantification of the other metabolites.

The analysis was performed on a HP 1100 high-performance liquid chromatography (HPLC) system (Hewlett-Packard, Meyrin, Switzerland) equipped with an injector loop of 100 μl. A chiral analytical column (Cyclobond I 2000, Acetylated β-cyclodextrin, Cat. No. 20124/Series No. 17514.250 × 4.6 mM from ASTEC (Whippany, NJ, USA) was coupled with a fluorimetric detector (Perkin-Elmer LC 240; Perkin-Elmer, Le Mont-sur-Lausanne, Switzerland) set at 236 and 304 nm for excitation and emission, respectively. The column flow rate was 0.6 ml/min, the mobile phase was methanol – citric acid buffer 0.005 mM/triethylamine (TEA) pH 6.3 (60/40 v/v). The mobile phase was slightly modified compared to the mobile phase described by Carlsson et al. (2001), Carlsson and Norlander (2001). Typical retention times were as follows: alprenolol: 8.022 min; *S*-DDCIT: 13.124 min; *R*-DDCIT: 14.395 min; *S*-DCIT: 16.894 min; *R*-DCIT: 18.649 min; *S*-CIT: 22.271 min; *R*-CIT: 24.267 minutes. Calibration curves were prepared to allow the stereoselective quantification of DCIT and DDCIT.

### Statistical and data analysis

The results were expressed as the means ± standard deviation (mean ± SD). For statistical calculations related to the inhibition study (e.g., Student *t*-test for dependent and independent means), the Statistica package (Statsoft v4.5; Loll and Nielsen, Germany) and SPSS (v26; IBM corporation, USA) were used.

Kinetic analyses were performed according to procedures described in a previous study (Rochat et al. 1997). Eadie-Hofstee representations (V against V/[S]) of the kinetic data were plotted to determine the presence of monophasic or biphasic models.

If a monophasic model of the Michaelis-Menten equation was observed, V_max_ and K_m_ were calculated with the following expression:

$$\text{v}={\text{V}}_{\max}\;[\text{S}]/({\text{K}}_{\text{m}}+[\text{S}])={\text{V}}_{\max}\;/(1+{\text{K}}_{\text{m}}/[\text{S}])$$ (1)

If a biphasic model of the Michaelis-Menten equation was observed, V_max1_, V_max2_ and K_m1_, K_m2_ were determined with the following expression:

$$\text{v}=\{{\text{V}}_{\max1}[\text{S}]/({\text{K}}_{\text{m1}}+[\text{S}])\}+\{{\text{V}}_{\max2}[\text{S}]/({\text{K}}_{\text{m}2}+[\text{S}])\}$$ (2)

The intrinsic clearances (ICs) were calculated as follows:

$$\text{IC}={\text{V}}_{\max}/{\text{K}}_{\text{m}}$$ (3)

V_max_, K_m_ and IC are expressed in pmol × min^–1^ × mg of microsomes proteins^–1^, μM and μl × min^–1^ × mg of microsomes proteins^–1^.

The results of the inhibition study are expressed as % of the control incubation, where 100% represents the production of DCIT or DDCIT in the absence of inhibitors.

## RESULTS

### Kinetic study

**Figure 1:**
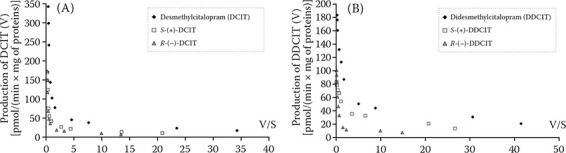
(A) Eadie-Hofstee plots of citalopram (CIT) and (B) desmethylcitalopram (DCIT) *N*-demethylation to DCIT and didesmethylcitalopram (DDCIT), respectively The plots underscore the existence of high affinity-low velocity (V) and low affinity-high velocity enzymes corresponding to, respectively, the left and right part of the plot. Concentration range of the substrates (S) CIT and DCIT: 0.5 μM to 1 000 μM

In Figure 1A,B, the *in vitro* biotransformation kinetics of both CIT and DCIT enantiomers to their respective *N*-demethylated metabolites DCIT and DDCIT in the Beagle dog liver microsomes are presented. The Eadie-Hofstee plots suggest the absence of linearity indicating that more than one isozyme is involved for both substrates and their enantiomers. The kinetic constants are expressed in Table 1. The first isozyme(s) involved in both the CIT and DCIT demethylation show extremely low K_m1_ values in contrast to the K_m2_ values. The K_m_ values range from 0.3 μM to 1.4 μM and from 66 μM to 1 221 μM, respectively. Thus, the first involved isozyme(s) show a very high affinity for CIT and DCIT enantiomers (K_m_ ≤ 1.4 μM) whereas the second involved isozyme(s) displays a poor affinity for the enantiomers of CIT (K_m_ ≥ 848 μM) and an intermediate affinity for the DCIT enantiomers (K_m_: 66 μM and 212 μM).

**Table 1:** Kinetic parameter values for the *in vitro* biotransformation of citalopram (CIT) and desmethylcitalopram (DCIT) to DCIT and didesmethyl-CIT, respectively in human and Beagle dog microsomes

Substrate	Metabolite	K_m1_	V_max1_	IC_1_		K_m2_	V_max2_	IC_2_	References
Dog liver microsomes*									
CIT	DCIT	1.1 ± 0.3	47 ± 3	43		1 006 ± 94	591 ± 28	0.6	this study
CIT	*S*-DCIT	1.0 ± 0.2	26 ± 1	27		848 ± 69	264 ± 10	0.3	
CIT	*R*-DCIT	1.4 ± 0.5	21 ± 2	15		1 221 ± 161	338 ± 24	0.3	
									
DCIT	DDCIT	0.6 ± 0.2	44 ± 4	73		137 ± 18	155 ± 5	1.0	this study
DCIT	*S*-DDCIT	0.6 ± 0.1	31 ± 3	52		66 ± 11	55 ± 2	0.8	
DCIT	*R*-DDCIT	0.3 ± 0.2	11 ± 2	37		212 ± 24	105 ± 3	0.5	
									
Human liver microsomes									
CIT	DCIT	174	–	–		–	–	–	von Moltke et al. (1999)
									
CIT	*S*-DCIT	33.8	20.0	0.6		202	220	1.09	Rochat et al. (1997)
CIT	*R*-DCIT	17.3	25.0	1.4		185	153	0.83	
									
CIT	*S*-DCIT	95	433	4.6		–	–	–	Olesen and Linnet (1999)
CIT	*R*-DCIT	76	300	3.9		–	–	–	
									
CIT	*S*-DCIT	165	1 007	6.1		–	–	–	von Moltke et al. (2001)
CIT	*R*-DCIT	256	1 254	4.9		–	–	–	
									
DCIT	*S*-DDCIT	72	95	1.3		–	–	–	von Moltke et al. (2001)
DCIT	*R*-DDCIT	108	126	1.2		–	–	–	

*Results are presented as means ± SD from six incubations carried out in duplicate

Intrisic clearence (IC) corresponds to the maximum velocity (V_max_) divided by the affinity constant, K_m_

IC = μl/(min × mg of protein); K_m_ = μM; V_max_ = pmol/(min × mg of protein)

Among the calculated IC-values (IC = V_max_/K_m_), those of IC_1_ show a very high biotransformation capacity of the implicated enzyme for both the CIT and DCIT enantiomers. In contrast, the considerably lower IC_2_ values indicate a much lower degradation capacity by some low affinity isozyme(s). As an example, the IC_1_/IC_2_ (CIT) ratios differ by a factor of 50 (*R*-DCIT: 15/0.3) and 90 (*S*-DCIT: 27/0.3), respectively, and the situation is similar regarding the metabolism of DCIT (Table 1). The data of Table 1 show some stereoselective differences in the *N*-demethylation of both substrates. For instance, the IC_1_ values of *S*-CIT and *S*-DCIT *N*-demethylation are somewhat higher than those of *R*-CIT and *R*-DCIT indicating that the *S*-enantiomers are more rapidly metabolised, but the design of this pilot study does not allow for a statistical analysis.

This limitation has also to be considered when the IC_1_ values of the biotransformation of CIT and DCIT are compared: The IC_1_ value calculated from the production of DDCIT from DCIT [73.0 μl/(min × mg proteins)] is higher than that concerning DCIT production from CIT [42.7 μl/(min × mg proteins)]. The situation is similar, when the kinetics of the individual enantiomers of CIT and DCIT are compared (Table 1).

### Inhibition study

The role of the different isozymes of CYP450 in the *N*-demethylation of CIT was studied by carrying out incubation experiments in the presence of inhibitors. In their absence, control incubations with 10 μM CIT showed a stereoselective production in favour of *S*-DCIT [50.6 ± 5.69 pmol/(min × mg protein)] rather than of *R*-DCIT [35.7 ± 9.06 pmol/(min × mg protein)] (*t* = 9.04; *P* ≤ 0.001; *n* = 5). After incubation with 100 μM CIT, the corresponding figures were 117 ± 10 pmol/(min × mg protein) and 104 ± 11.6 pmol/(min × mg protein) (*t* = 2.54; ns; *n* = 5), respectively.

Similarly, control incubations with 10 μM DCIT showed a stereoselective production in favour of *S*-DDCIT [199 ± 7.4 pmol/(min × mg protein)] rather than of *R*-DDCIT [78.6 ± 2.80 pmol/(min × mg protein)] (*t* = 45.18; *P* ≤ 0.001; *n* = 3), while after incubation with 100 μM DCIT, the corresponding figures were 275 ± 7.7 pmol/(min × mg protein) and 240 ± 5.9 pmol/(min × mg protein) (*t* = 32.17; *P* ≤ 0.001; *n* = 3), respectively.

These observations, in the absence of inhibitors, are in line with the results of the kinetic study that show more rapid *N*-demethylation of *S*-CIT and *S*-DCIT than that of the *R*-enantiomers.

**Figure 2:**
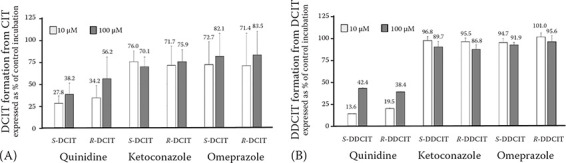
Inhibition of the citalopram (CIT) and desmethylcitalopram (DCIT) demethylation by three different chemical inhibitors. The results are expressed as % [the numbers present the means (± SD)] of the control activity (set at 100%) (A) CIT to DCIT (mean of 5 experiments) and (B) DCIT to DDCIT (mean of 3 experiments). The substrate concentrations were 10 and 100 μM. The inhibitors quinidine (100 μM), ketoconazole (1 μM) and omeprazole (100 μM) were used at concentrations at least partly selective for 2D15, 3A12 and 2C21/41, respectively

Figure 2A shows the percentual (mean ± SD) formation of DCIT from CIT, in the presence of different inhibitors in comparison to the control experiments. After the addition of 100 μM of quinidine to 10 μM CIT, the difference in the production of *S*-DCIT from CIT, in comparison to that in the absence of the inhibitor, was larger than that concerning *R*-DCIT: 36.9 ± 8.53 pmol/(min × mg protein) vs. 24.5 ± 11.4 pmol/(min × mg protein) (*t* = 8.87; *P* ≤ 0.01; *n* = 5). Therefore, quinidine inhibited the *S*-DCIT production more potently than that of *R*-DCIT. Depending on the dose of CIT, the mean inhibition of the formation of its demethylated metabolite by the CYP2D15 inhibitor quinidine reached between 43.8% and 72.2% (Figure 2). The differences mentioned above were not significant when other inhibitors were considered: The CYP3A12 inhibitor ketoconazole (1 μM) and the CYP2C21/41 inhibitor omeprazole (10 μM) inhibited the *N*-demethylation of CIT by only 24.1–29.1% and 16.5–28.6%, respectively (Figure 2).

Figure 2B presents the formation of DDCIT from DCIT, in the presence of different inhibitors in comparison to the control experiments. After the addition of 100 μM quinidine to 10 μM DCIT, the difference of the production of *S*-DDCIT from CIT, in comparison to that in the absence of the inhibitor, was larger than that concerning *R*-DDCIT: 172 ± 6.6 pmol/(min × mg protein) vs. 63.3 ± 2.72 pmol/(min × mg protein) (*t* = 46.9; *P* ≤ 0.000 1; *n* = 3).

The inhibition reached 86.4% (at 10 μM DCIT) and 57.6% (at 100 μM DCIT), regarding the *S*-DDCIT production, while for *R*-DDCIT, 80.5% and 61.6% inhibition was measured (Figure 2B), respectively. On the other hand, the CYP3A12 inhibitor, ketoconazole, and the CYP2C21/41 inhibitor, omeprazole, inhibited the *N*-demethylation of DCIT (10 μM, 100 μM) by less than 15% (Figure 2). The other inhibitors, methylpyrazole, sulphaphenazole and furafylline, did not notably modify the production of the DCIT and DDCIT enantiomers.

## DISCUSSION

Authors from the manufacturer of CIT (Boeck et al. 1982; Fredricson Overo 1982; Fredricson Overo 1989; Fredricson Overo and Hojelse 1994) reported convulsions in Beagle dogs after CIT was administered to these animals. Prior to the present pilot study on the stereoselective metabolism of CIT to DCIT and DCIT to DDCIT by CYP450, no *in vivo* or *in vitro* investigations dealt with the elucidation of the enzymatic mechanisms involved in their biotransformation in dogs. This scarcity of studies is surprising, as the clinical development was delayed for several years in the mid-eighties due to the death of some animals after a 5- to 6-month CIT treatment in a 12-month dog toxicity study (Fredricson Overo and Hojelse 1994). The following statement of the FDA summarises the situation: “Although appropriate data are not available to directly compare the plasma levels of CIT and its metabolites, DCIT and DDCIT, to levels that have been achieved in humans, the pharmacokinetic data indicate that the relative dog-to-human exposure is greater for the metabolites than for citalopram” (FDA 2017). As dogs are used as a valuable species for the estimation of toxicological drug properties in humans, not only comparative studies about the pharmacokinetics, but also role of enzymes in the metabolism of CIT and DCIT are necessary.

The present data about the *N*-demethylation of CIT to DCIT in dog liver microsomes (Figure 1A, Table 1) reveal the implication of a very high affinity enzyme (K_m1_: 1.1 μM) as well as other cytochrome P-450 isoforms with a much lower affinity (K_m2_: 1 006 μM). The calculated IC_1_ and IC_2_ values demonstrate an important difference in the clearance capacity of the very high and the poor affinity enzymes. In contrast, previous *in vitro* kinetic studies about the stereoselective CIT *N*-demethylation performed with human liver microsomes did not show CYP450 isozymes with such a high affinity and high clearance capacity: the K_m_ values were ≥ 17.3 μM and the IC values below ≤ 6.1 pmol/(min × mg protein) (Table 1) (Rochat et al. 1997; Olesen and Linnet 1999; von Moltke et al. 2001). Therefore, for this metabolic step, the isozyme affinity is about 10 times higher in Beagle dogs and, more importantly, the IC is also about 10 times higher in Beagle dog than in humans.

Similarly, for the *N*-demethylation of DCIT enantiomers in humans, the K_m_ values were ≥ 72 μM and the IC values below ≤ 1.3 pmol/(min × mg protein) (Figure 1B, Table 1) (von Moltke et al. 2001). Therefore, for the *N*-demethylation of DCIT enantiomers, the isozyme affinity is at least 100 times higher in Beagle dogs, while the IC is about 30 times higher in Beagle dogs than in humans.

In Table 1, the production of DCIT from CIT in human microsomes appears to be higher than that of DDCIT from DCIT (von Moltke et al. 2001), while in dog microsomes, the situation is inverse, as shown in the present study. This is in line with the *in vivo* pharmacokinetic data obtained after administration of CIT to patients. Indeed, the DDCIT plasma concentrations are considerably lower than those of DCIT in humans (Rochat et al. 1995; Sidhu et al. 1997; Rasmussen et al. 1999; Carlsson et al. 2001; Le Bloc’h et al. 2003), while much higher plasma concentrations of DDCIT in comparison to DCIT, are measured in dogs after the administration of CIT (Fredricson Overo 1982; Matsui et al. 1995).

Some of the following discussion deals with the question, whether and to which extent some forms of human and dog P450 present share orthologs and then display similar properties towards substrates and inhibitors. It is to be stated that the CYP450 isoforms in humans and dogs are not strictly identical, neither by their protein sequence, nor by their affinities for substrates. The human and canine genes are not clear orthologues (Bogaards et al. 2000; Shou et al. 2003; Martignoni et al. 2006; Turpeinen et al. 2007; Court 2013; Martinez et al. 2013; Sakai et al. 2014), and they also differ by their genetic polymorphisms (Martinez et al. 2013; van Hagen et al. 2020; Karakus et al. 2021). Similarly, selective inhibitors of human P450 isoforms may not display similar selective properties in dog microsomes (Martinez et al. 2013).

Pharmacokinetic and pharmacogenetic studies in humans demonstrated the involvement of CYP2C19 and CYP2D6 in the *N*-demeth-ylation of CIT to DCIT (Sindrup et al. 1993; Herrlin et al. 2003). CYP2C19 preferentially demethylates *S*-CIT rather than *R*-CIT (Herrlin et al. 2003; Baumann et al. 2021). Additional *in vitro* studies using human liver microsomes described the role of CYP3A4 in addition to CYP2C19 and CYP2D6 in this demethylation step (Kobayashi et al. 1997; Rochat et al. 1997; Olesen and Linnet 1999; von Moltke et al. 1999; von Moltke et al. 2001).

In the present study, quinidine caused the strongest inhibition of DCIT production in the dog liver microsomes suggesting that the canine orthologue of CYP2D6, i.e., CYP2D15 is involved in the *N*-demethylation of CIT (Figure 2). Quinidine preferentially inhibited the formation of *S*-DCIT rather than of *R*-DCIT. This strongly suggests that CYP2D15 is the isozyme with very high affinity and high IC capacity for *N*-demethylation of *S*- and *R*-CIT. CYP2D15 and CYP2D6 seem to share homologies with regard to their activity towards CIT *N*-demethylation (Olesen and Linnet 1999; von Moltke et al. 1999). However, in the present study, the concentration of quinidine chosen according to Chauret et al. (1997) was relatively high (100 μmol/l), possibly too high, and it cannot be excluded that other forms of cytochrome P-450 were also inhibited.

In comparison to quinidine, the CYP3A12 inhibitor ketoconazole (1 μM) and the CYP3A12 inhibitor omeprazole (10 μM) also decreased the DCIT production, but the contribution of these isozymes seems to be smaller than that of CYP2D15 (Figure 2). This suggests that both CYP3A12 and CYP2C21/41 are also involved in the *N*-demethylation of CIT in dog liver microsomes, but to a much lesser extent than CYP3A4 and CYP2C19, respectively, in human liver microsomes (Rochat et al. 1997; Olesen and Linnet 1999).

The other inhibitors, methylpyrazole, sulphaphenazole and furafylline, did not notably modify the production of DCIT and DDCIT enantiomers, suggesting that no other CYP450 isozymes other than CYP2D15, CYP3A12 and CYP2C21 were significantly involved (not shown).

It is not quite clear, whether other CYP450 forms also play a role, as seemingly there is a lack of corresponding studies. The results of the inhibition presented in Figure 2 strongly suggest the involvement of CYP2D15 as the very high affinity and high clearance capacity isozyme involved in the biotransformations of CIT to DCIT and of DCIT to DDCIT in the dog liver. The CYP450 isozymes with low and moderate affinities and low clearance capacities involved in the same biotransformation should be CYP3A12 and CYP2C21/41 because minor inhibitions were observed.

There are several limitations of this investigation which need to be mentioned. The liver of only one dog which, moreover, was not genotyped, was used to prepare the microsomes and was submitted to the pharmacological investigation. For the inhibition study, only a few inhibitors were used, and while they present some selectivity in investigations with human liver preparations (Chauret et al. 1997; Martinez et al. 2013), this is not the case with those prepared from dog livers, as already discussed above. To ascertain the role of particular P450 isoforms in the metabolism of CIT and DCIT, studies with additional inhibitors, but also with inducers, should be carried out. In addition, screening recombinant P450 enzymes would be a valuables approach.

**Figure 3:**
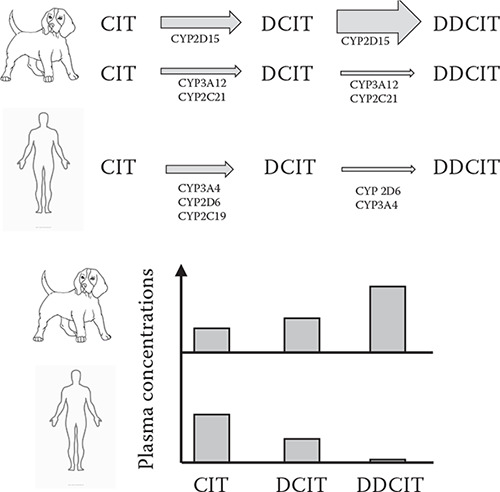
Comparative metabolism and pharmacokinetics of citalopram (CIT) in the human and Beagle dog (A) Citalopram (CIT) *N*-demethylation pathways to desmethyl-CIT (DCIT) and didesmethyl-CIT (DDCIT) in man and in Beagle dog liver microsomes according to the presented kinetic and inhibition studies. Arrow widths relate to the clearance of the pathways (IC = V_max_/K_m_ ratios). (B) Schematic comparison of plasma levels in dogs (Matsui et al. 1995; Fredricson Overo 1982) and in man (Rochat et al. 1995; Sidhu et al. 1997; Rasmussen et al. 1999; Carlsson et al. 2001) after CIT exposition, as reported in the literature. Stereoselectivity in the pathways is not depicted because the consequences are minor

Nevertheless, taking the *in vivo* pharmacokinetic studies realised in dogs after CIT exposure into account (Boeck et al. 1982; Fredricson Overo 1982; Fredricson Overo 1989; Fredricson Ove-ro and Hojelse 1994), the *in vitro* experiments of this present study suggest, to some extent, that the canine CYP2D15, which presents some analogy with the human CYP2D6, plays a major role in the *N*-demethylation of CIT and especially of DCIT. This should explain the considerably higher formation rate of DDCIT in dogs in comparison to humans where CYP2D6 plays a modest role in the formation of DCIT and DDCIT, but clearly, further studies are needed with appropriate inhibitors. The intrinsic clearance (*in vitro*) and the *in vivo* plasma levels of CIT, DCIT and DDCIT are schematically depicted for the dog and humans in Figure 3. In this comparative situation, the dog animal model seems to have a limited predictive value for the human plasma levels of CIT, DCIT and DDCIT and the toxicity related to DDCIT at a plasma level ≥ 1 000 nM (300 ng/ml) (Shanks et al. 2009). Indeed, in a therapeutic drug monitoring study, where several patients were medicated with CIT doses between 60 and 360 mg/day, the DDCIT plasma concentrations only reached between 40 ng/ml and 100 ng/ml (Le Bloc’h et al. 2003). They are well below a range which was considered cardiotoxic in dogs.
